# Post-Fermentation Application of Pea Protein-Based Fining Agents: Effects on Aromatic White Wine from *Tămâioasa Românească*

**DOI:** 10.3390/foods14193448

**Published:** 2025-10-09

**Authors:** Oana Arina Antoce, George Adrian Cojocaru

**Affiliations:** Department of Bioengineering of Horti-Viticultural Systems, Faculty of Horticulture, University of Agronomic Sciences and Veterinary Medicine of Bucharest, 59 Marasti Ave., Sector 1, 011464 Bucharest, Romania; george.cojocaru@usamv.ro

**Keywords:** pea protein, yeast hulls, chitosan, bentonite, active carbon, wine fining agents, gas chromatography, electronic nose, volatile profile, aroma profile

## Abstract

Pea protein is increasingly used as a plant-based alternative for fining white wines, aiming to reduce excessive polyphenols while replacing animal-derived or synthetic agents such as PVPP. This study compared pea protein alone (P), PVPP (PV), and untreated control wines (V0) with five combinations containing pea protein and additional agents, such as activated carbon (C), bentonite (B), yeast hulls (Y), and fungal chitosan (K), forming the variants PCB, PYB, PCY, PKY, and PKC applied in doses of 20 g/hL. Fining was applied to aromatic white wines of *Tămâioasa Românească* in triplicate (50 L tanks), obtained and followed by standard vinification steps. Main wine parameters (ethanol, malic acid, acetic acid, pH) were largely unaffected by the treatments, while free sugar levels showed only slight variations. Some significant differences were observed in total acidity. Total polyphenol content was significantly reduced by ternary fining combinations containing pea protein and yeast extract (PCY and PKY), as well as by PVPP, with reductions of approximately 37% compared to the control. Proanthocyanidins were largely preserved irrespective of the treatment, whereas flavan-3,4-diols were significantly reduced by PVPP. The fining treatments induced only small, imperceptible differences in colour, detectable solely through CIELab measurements, with the classical PVPP treatment producing wines with wines with the greenest colour tones. Volatile profiles, assessed using a GC analyser with two columns (Heracles electronic nose), were analysed in detail. Of all the 8 experimental variants, chitosan- and yeast hull-containing combinations (PKY and PCY) enhanced both varietal and fermentation-derived aromas, particularly terpenes and key esters, producing the most expressive and complex wines. In these variants, compared to control wines, eucalyptol, linalool, and *trans*-linalool oxide increased approximately by 13.1–23.2%, 16.7–19.3% and 341.5–428.7%, respectively. Pea protein alone preserved the aroma profile closest to the untreated control, inducing no significant differences in all the compound classes, making it a suitable alternative to PVPP. In contrast, bentonite-containing treatments reduced ester and terpene concentrations, simplifying the aroma profile and diminishing varietal characteristics. In bentonite variants, especially PCB, reduced key aroma compounds such as 2-phenylethyl acetate by 17.9%, ethyl octanoate by 12.9% and ethyl decanoate by 33.0%, linalool and *trans*-linalool oxide, by 18.5%, and 22.7%, respectively. These results support the use of pea protein as a selective and minimally disruptive fining agent, suitable for reducing polyphenol content while preserving wine quality. Pea protein combinations with yeast hulls, and to some extent with chitosan or other fining agents, can further enhance aroma complexity and varietal expression.

## 1. Introduction

Fining treatments are an essential part of winemaking, widely applied to eliminate undesirable components and thereby improve wine quality. These interventions consist of adding specific processing aids at different stages of vinification in order to precipitate targeted particles. In doing so, they contribute to wine clarity, physicochemical stability, and refinement of sensory attributes. Among the many objectives of fining, one of the most critical is the reduction of excessive phenolic compounds. This is particularly important in white wines, where phenolics are prone to oxidation, accelerating browning, and contributing to bitterness and astringency, sensory effects that significantly compromise consumer acceptance.

Traditionally, polyvinylpolypyrrolidone (PVPP) has been considered the most effective agent for phenolic removal. However, PVPP is a synthetic polymer and, as such, is poorly perceived by certain consumer groups. Similarly, animal-derived proteins such as gelatin, albumin, egg whites, casein, skim milk, and isinglass have been used with considerable efficiency but face increasing resistance as they also raise concerns [1,2]. A major drawback of animal protein agents is their allergenic potential [3], due to which their pre-sence in foods must be declared on wine labels [4,5]. In response, research and industry have increasingly turned toward vegetal protein-based and other non-proteic natural agents. Several of these are already recognized and authorized by the OIV, including pea, potato and cereal proteins [6], as well as chitosan and chitin-glucan [7,8] and yeast hulls [9,10]. Other emerging alternatives include rice and soy proteins [11], grape pomace [12], as well as algae proteins [13]. The growing availability of such alternatives reflects not only technological innovation, but also a shift toward sustainability, allergen safety, and consumer acceptance.

The effectiveness of these agents replacing PVPP or animal protein-based agents is now well supported by evidence. Many studies confirm their ability to significantly reduce phenolic content and turbidity [2,11,14,15,16]. Within this group, pea and potato proteins are especially valued, because they combine fining efficiency with a substantially lower allergenic risk in adults, although reactions have been reported in children [17]. Moreover, these proteins demonstrate selective precipitation of tannins and effective stabilization of both white and red wines [15,18].

Still, the use of alternative fining agents is not without challenges. Although several reports indicate minimal effects on aroma [1,2,11,14,15,16], other studies provide clear evidence that some of these agents, while removing unwanted compounds, can also strip volatile aroma molecules, reducing the sensory quality of wines [10,14], thus counterbalancing the beneficial effects. This trade-off represents one of the central obstacles in replacing traditional fining agents and underlines the need for fine-tuned strategies.

To overcome this limitation, research must prioritize fining agents with mild sensory impacts, or seek synergistic formulations that combine different materials to balance efficiency and preservation of aroma. In this regard, pea protein emerges as a particularly promising solution [19]. Not only is it sustainable, effective and safe, but it also lends to combination with other agents such as chitosan, yeast hulls, bentonite or activated carbon, offering the possibility of tailored ternary systems with counterbalancing or synergistic properties. Building on previous research highlighting the importance of selectively removing excessive polyphenols, this study aims to demonstrate that pea protein, either alone or in combination with complementary fining agents, can effectively maintain wine colour while enhancing the aromatic profile, compared to PVPP. Indeed, our previous experiments with pea protein-based ternary combinations in must showed encouraging results [10], although in some cases volatile compounds responsible for aroma were reduced, leading to perceivable sensory effects [20].

For these reasons, the present study focuses on post-fermentation fining treatments applied directly in wine, where a more controlled evaluation of polyphenols, colour, and volatile aroma compounds is possible. Moreover, the study was conducted on an aromatic grape variety, since such wines are especially sensitive to fining agents, which may remove essential volatile compounds responsible for their distinctive typicity. Nevertheless, research on these varieties remains limited. The variety chosen for this research is *Tămâioasă Românească*, a highly aromatic Romanian Muscat-type grape also known as *Busuioacă de Moldova*, *Muscat Blanc à Petits Grains*, *Rumanische Weihrauchtraube*, *Tamianka*, and *Tămâioasă Albă de Drăgășani* [21,22]. This variety is an ideal model, as it is typically vinified with maceration, a process that enhances aroma extraction, but also increases phenolic load, thus intensifying the challenge of balancing fining effectiveness with aroma preservation. The sensory typicity of *Tămâioasă Românească* is largely defined by terpenoids and ethyl esters [10,23]. Consequently, any fining strategy applied to this variety must achieve a delicate balance: it must effectively remove excess oxidizable phenolics while simultaneously preserving the volatile compounds responsible for its characteristic aroma profile. By addressing this dual requirement, the present work seeks to provide new insights into how innovative fining agents, particularly those based on pea protein, can be optimized to ensure wine stability and quality, while also responding to modern expectations of safety, sustainability, and consumer acceptance.

## 2. Materials and Methods

### 2.1. Materials: Wine and Fining Agents

Wine from the *Tămâioasă Românească* aromatic variety, produced in a large, uniform industrial batch at the Pietroasa Research Station of the University of Agronomic Sciences and Veterinary Medicine of Bucharest, was used for the experiments. A fraction of 1000 L of this untreated wine was extracted from a 10,000 L tank and transported under controlled conditions to USAMVB, where fining treatments were applied in stainless steel tanks with a volume of 40 L (8 variants, each in triplicate). As the experiments aimed to remove polyphenols from the wine samples, the *Tămâioasă Românească* wine used also contained fractions obtained from pressing.

The substances used for preparing the solutions for fining are the following: SMARTVIN PVPP (Enologica Vason, Settimo, Italy), pea protein Proveget (Agrovin, Ciudad Real, Spain), chitosan Kitosmart (Enologica Vason, Settimo, Italy), yeast cell walls OENOLEES (Laffort, Floriac, France), active carbon Acticarbone (2SW, CECA Arkema, Lannemezan, France), and Ca-bentonite Microcol CL G (Laffort, Floriac, France).

### 2.2. Experiments for Wine Fining

The experimental wine samples were prepared in triplicates in stainless steel tanks of 50 L volume, in which 40 L of wines were introduced and cooled to 17 °C before treatment. Control wines (V0) were not treated with any fining agents and only kept in the same conditions as the rest of the wines. The classical fining treatment for polyphenol removal was applied in tanks PV_1 to PV_3, by adding a dose of 20 g/hL PVPP. Similarly, the alternative treatment was applied in tanks P_1 to P_3, by adding a dose of 20 g/hL pea protein. Other 5 alternative ternary combinations based all on pea protein were used for the samples coded with 3 letters, all in triplicate. For the ternary combinations the total fining dose was kept the same, 20 g/hL, only the pea protein was reduced to 50% (10 g/hL) and other two fining agents were introduced, in doses of 5 g/hL each. The complementary fining agents used along with the pea protein were selected from the following substances: calcic bentonite (B), activated carbon (C), yeast hulls (Y) and chitosan (K). The materials and doses used for the experimental wine samples are summaries in Table 1.

The total dosage of 20 g/hL for all fining treatments was selected taking into account that this is the minimum dose for many approved fining agents. The goal of this study was to obtain a sufficient effect as far as removing polyphenols, but also intervene as little as possible on the fine chemical balance of wine and provide the oenologists with cost-effective fining agents. Therefore, the minimum approved and demonstrated efficient doses were tested for all the fining agents selected in the study. The total dosage of pea protein was based on the minimum dose recommended by the producer (Agrovin, Ciudad Real, Spain), which advises treatments within the 20–50 g/hL range, the upper limit of 50 g/hL also being stipulated by the OIV International Code of Oenological Practices [24]. Also, in accordance with OIV [24], PVPP treatments cannot exceed 80 g/hL and are generally recommended within the 20–80 g/hL range, making 20 g/hL the minimum dose with proven efficacy. For consistency and comparability, all treatments, including those with ternary combinations, were applied at a total dosage of 20 g/hL, with complementary fining agents in the combinations replacing the corresponding quantity of pea protein.

The proportions in the ternary combinations were tested in our previous researches, including on experiments performed in musts [10] and were protected by filling a patent in the beginning of 2025 [25]. The doses for all materials, administered either individually or as part of ternary combinations, are detailed in Table 1.

Forty-eight hours after treatment, the wines are racked off the sediment and then bottled in 0.75 L bottles. One month after bottling, the wines are subjected to analysis.

The individual fining agents were prepared as 10% solutions, and the appropriate volumes to achieve the desired doses were pipetted and thoroughly mixed into the wines, which were kept in thermostated stainless steel tanks at 20 °C. For ternary combinations, the order of addition followed that specified in Table 1. One month after treatment, the wines were racked from the sediment formed by the fining agents and subsequently analysed.

### 2.3. Wine Analysis

#### 2.3.1. Main Wine Parameters Determinations

The main parameters of wines were determined in accordance to the methods recommended by the International Organisation of Vine and Wine and included in the Compendium of International Methods of Wine and Must Analysis [26].

Ethanol content (expressed as alcoholic strength by volume, % *v*/*v*) was determined using the OIV pycnometric method comprised in the Compendium as OIV-MA-AS312-01 Alcoholic strength by volume [26].

Enzymatic assays with absorbance readings at 340 nm were employed to determine free sugars (expressed as D-glucose + D-fructose, g/L), malic acid (g/L), and acetic acid (g/L), using the methods OIV-MA-AS311-02 Glucose and fructose, OIV-MA-AS313-11 L-Malic acid, and OIV-MA-AS313-27 Determination of acetic acid in wines by automated enzymatic method comprised the OIV Compendium [26].

Colorimetric methods were used to measure total SO_2_ (expressed as total sulphites, mg/L), pH, and titratable acidity (g/L), with absorbance readings at 405 nm, 600 nm, and 620 nm, respectively. The methods for sulphites determination in wines is based on the reaction of sulphur dioxide (SO_2_) with 5,5′-dithio-bis-(2-nitrobenzoic acid) (DTNB) in an alkaline medium (pH 8.2), producing the yellow thiol 5-mercapto-2-nitrobenzoate, which is quantified by measuring absorbance at 405 nm. The alkaline conditions ensure the release of bound sulphur dioxide from the wine sample and convert hydrogen sulphite into sulphite [27]. For total acidity, the colorimetric method employed was based on the classical principle that acids in the sample alter the pH of the reaction mixture. In the presence of the bromothymol blue (BTB) indicator, these changes can be quantified spectrophotometrically. Similarly, the pH can also be determined using the colorimetric bromothymol blue indicator [28].

Polyphenols in the sample react with Folin–Ciocalteu’s reagent in an alkaline medium. The resulting increase in coloration is proportional to the polyphenol concentration in the sample. The reaction forms a polyphenol–FC complex, which could be quantified at the wavelength of 750 nm [29,30].

The enzymic and colorimeter assays were performed with an multiparametric analyser Y15 from BioSystems (Barcelona, Spain), using a halogen lamp 6 V/10 W as light source, a silicone photodiode as photometric detection system and filters for the wavelengths 340, 405, 420, 520, 560, 600, 620, 635, 670 and 750 nm, in combination with BioSystems reagent kits [31,32].

To quantify proanthocyanidins (condensed tannins) and flavan-3,4-diols (leucoanthocyanidins) present in wine, a modified method [33,34] based of the original method described by Bate-Smith [35], generally known as the acid butanol assay, was used. This method relies on the acid-catalysed cleavage of flavan-3,4-diols at room temperature and of proanthocyanidins upon heating, in the presence of a butanol–HCl reagent, prepared by dissolving in a 500 mL mixture of *n*-butanol/HCl (3:2, *v*/*v*) of 77 mg/L FeSO_4_·7H_2_O, which enhances colour development. The reaction produces red-coloured anthocyanidins, which are measured spectrophotometrically at 550 nm. For the assay, two test tubes are prepared by mixing 0.5 mL of wine with 5 mL of the butanol–HCl reagent. One tube is left at room temperature to measure flavan-3,4-diols, while the other is incubated in a boiling water bath (95 °C) for 50 min to measure the combined content of flavan-3,4-diols and proanthocyanidins. After cooling, the absorbance of both solutions is measured at 550 nm using a Specord 250 spectrophotometer (Analytik Jena, Jena, Germany). The final results are expressed as mg L^−1^ of cyanidin equivalents, calculated according to the Lambert–Beer law, based on the molar mass of cyanidin and its molar absorptivity coefficient (ε) of 34,700 L·mol^−1^·cm^−1^ at 550 nm.

#### 2.3.2. Wine Colour Determination

Colour assessment was also performed by spectrometry, glass cuvettes of 1 cm pathlength being used for visible spectrum determinations and calculations of the CIELab coordinates. Conversion of absorbance spectra to CIELab coordinates was done using standard illuminant D and observer angles 10°. CIELab data acquisition and analysis were performed using WinAspect software, version 2.2.7 (Analytik, Jena, Germany). The CIELab parameters and their significance have been described in detail in previous publications [36,37]. In brief, the CIELab colour space is defined by three coordinates: L (lightness, ranging from 0 for black to 100 for white), *a* (position between green and red, with negative values indicating green and positive values indicating red), and *b* (position between blue and yellow, with negative values indicating blue and positive values indicating yellow). Based on *a* and *b* parameters, other two are calculated: (chroma), is a measure of colour intensity or saturation (higher values indicating more vivid, intense colours; lower values duller, less saturated colours) and *h* (Hue angle) which represents the dominant colour tone, expressed in degrees around the CIELab colour circle (0° → red, 90° → yellow, 180° → green, 270° → blue).

The total colour difference (ΔE) was calculated according to the formula:

$$∆E=\sqrt{{\left(L_{c}-L_{s}\right)}^{2}+{\left(a_{c}-a_{s}\right)}^{2}+{\left(b_{c}-b_{s}\right)}^{2}}$$

where subscripts c and s represent the control and sample, respectively.

In practical terms, a ΔE value of less than 1 is generally not perceptible to the human eye, values between 1 and 3 are perceptible only to trained observers, and values above 3 are considered clearly noticeable colour differences [38,39].

#### 2.3.3. Wine Volatile Compounds Evaluation

The volatile components of the wine variants were analysed using the Heracles e-Nose gas chromatograph (Alpha MOS, Toulouse, France), equipped with two short capillary columns of differing polarity: a non-polar DB5 column (5% diphenyl, 95% dimethylpolysiloxane) and a low-to-mid polarity DB1701 column (14% cyanopropylphenyl, 86% dimethylpolysiloxane). This setup enables rapid separation of the main aroma compounds. Samples were prepared by extracting 200 μL of headspace gas from 10 mL vials containing 4 mL of wine, sealed with magnetic caps, following a method developed in our laboratory [40,41]. The instrument’s Tenax trap facilitates pre-concentration of volatile organic compounds prior to injection.

Detection was carried out by flame ionization detectors (FIDs) placed at the end of each column, with high purity hydrogen serving as both the carrier gas and combustion gas for the FIDs. Each of the eight wine variants and their repetitions was analysed in triplicate, totalling 72 GC runs. The resulting chromatograms were processed using AlphaSoft v12.42 software, which controls the autosampler and records data from both columns simultaneously.

Identification of volatile compounds was based on average chromatographic peak areas and Kovats retention indices, using the Heracles apparatus chemical database, AroChemBase (Toulouse, France: Alpha MOS; version 2010), supplemented with external databases including Pherobase [42], Flavornet [43], and NIST [44].

Further methodological details, chromatogram examples, sensor selection procedures, and Kovats index calibration are available in previous publications [40,41,45,46,47,48].

For each identified compound, the weighted retention time (Weighted RT) and adjusted coefficient of determination (Adj. R^2^), were provided. Aroma descriptors were sourced from the mentioned databases, our previous results, and scientific literature.

### 2.4. Statistical Analysis

For the comparison of total chemical and CIELab parameters, the analysis of variance (one-way ANOVA) and the Tukey’s HSD test were applied using Origin Pro 2024 software package (OriginLab, Northampton, MA, USA). Levene’s test was applied to confirm that the variance across different groups was equal and for post-hoc pairwise comparison Tukey’s HSD (Tukey’s Honest Significant Difference) test was selected because it confers and excellent control over Type I error when it comes to comparisons of more than 3 groups.

For the e-nose data records, which contained some missing values due to the type of chromatographic columns used, Welch’s ANOVA was applied as it is more robust to violations of homogeneity of variances. This was followed by post hoc comparisons using the Games-Howell test, which accommodates unequal variances and unequal sample sizes. These tests were performed with SPSS version 26 (IBM Corp., Armonk, NY, USA).

Chromatographic data were processed using Origin 2024 software, which facilitated the generation of multivariate analyses including Principal Component Analysis (PCA), cluster analysis and heatmap plots. These visualization tools aided in identifying patterns, grouping similar samples, and highlighting differences in the volatile profiles of the wine variants.

## 3. Results

To evaluate the impact of the fining treatments on wine composition and quality attributes, a comprehensive set of analytical determinations was performed one month after treatment application.

### 3.1. Physicochemical Characteristics of the Experimental Wines

The main physicochemical parameters were determined to monitor potential changes in the basic composition of the wines, including ethanol content, residual sugars, and acidity-related indices, as these represent key indicators of stability and overall quality. The results are presented in Table 2, which summarizes the principal chemical parameters measured in the experimental wines.

Colour characteristics were determined in the CIELab space, providing an objective description of visual attributes and enabling the detection of even subtle changes in hue, chroma, and lightness (Table 3).

In parallel, the concentrations of total polyphenols and selected polyphenol classes were quantified (Table 4), as these compounds are closely associated with wine colour, flavour, and antioxidant capacity. Their assessment was of particular importance in this study, given that the primary objective was to reduce their levels in order to improve palatability and enhance the wines’ oxidative stability.

### 3.2. Volatile Profile of the Experimental Wines

Aside from the primary goal of these treatments, which is to partially remove undesirable polyphenols from the wines, preserving or enhancing the aromatic profile is equally important. For this purpose, a comprehensive evaluation of the volatile compounds, detectable by flash GC using two columns of different polarity, was performed to assess the impact of the proposed treatments on the aroma of the final wines. For substances identified on both columns, results from both columns are included. The reported Weighted Kovats Index (WKI) indicates compound identity based on retention time, accounting for variations between columns or experimental conditions, and provides a more accurate comparison to reference values than the Kovats Index generated by the Heracles analyser. Adj. R^2^ (Adjusted R^2^) shows the goodness of fit for peak quantification, values close to 1 indicating a very good fit, meaning the reported peak areas reliably represent the concentrations of the compounds.

## 4. Discussion

### 4.1. Influence of the Fining Treatments on the Main Wine Parameters

The fining treatments applied to reduce excessive polyphenols, preserve the quality of white wines and prevent oxidative deterioration had only minimal effects on the main chemical parameters of the treated wines.

As shown in Table 2, ethanol, malic acid, and acetic acid concentrations were not significantly affected by the treatments. Free sugar levels showed slight variations, decreasing from 2.22 g/L in the control samples to 2.03 g/L in the PVPP-treated wines, and to 1.68–1.96 g/L in the fined wines. This reduction is likely due to the clarifying and adsorptive effects of the fining agents, particularly those containing pea protein. Although this small decrease in sugar content had no measurable impact on the alcoholic strength of the wines, it may improve microbiological stability, since wines with residual sugar levels above 2 g/L remain susceptible to spoilage microorganisms [48].

Significant differences were observed in total acidity, with the lowest values recorded in the control wines. An increase in acidity was particularly evident in treatments with ternary fining combinations containing both pea protein and chitosan (PKY and PKC), where values rose from 4.79 g/L in the control to 4.97–4.98 g/L (a 23.8% increase). Other ternary combinations incorporating activated carbon (PCB and PCY) also showed increased acidity after treatments, up to 4.92 g/L. Although relatively small, these increases in titratable acidity may enhance wine stability, both in terms of colour and microbial spoilage resistance [49].

The pH values, ranging between 3.6 and 3.7, were essentially unaffected, despite statistical analyses indicating minor significant differences. Total sulphur dioxide, added during vinification for its antioxidant role, remained well below the maximum legal limit for dry white wines (200 mg/L) [50].

### 4.2. Influence of the Fining Treatments on the Wine Colour and Polypehnol Content

CIELab analysis was used to detect even subtle differences in the colour parameters of the wines, although visually the colour appeared unaffected by the fining treatments when assessed one month after their application. The measured colour parameters are presented in Table 3.

All samples exhibited excellent clarity, as indicated by the luminance (*L*) values, which ranged from 97.8 to 98.6 out of 100. The lowest clarity was observed in the PVPP-treated samples, which was significantly lower than the limpidity achieved with pea protein and its combinations.

The parameter *a*, representing the colour axis from green (negative values) to red (positive values), indicated that all samples fell within the green region of the colour space. The PVPP and PCY treatments produced the most intense green tones (*a* = −1.37 and −1.38, respectively), whereas the PYB-treated wine exhibited the least green tones (a* = −1.11).

The *b* parameter, representing the position of the colour between blue and yellow, indicated that all samples fell within the yellow region of the colour space (positive values). The yellowest tones were observed in the control sample (*b* = 7.5), whereas the least yellow tones were detected in PYB (*b* = 6.78).

**Figure 1:**
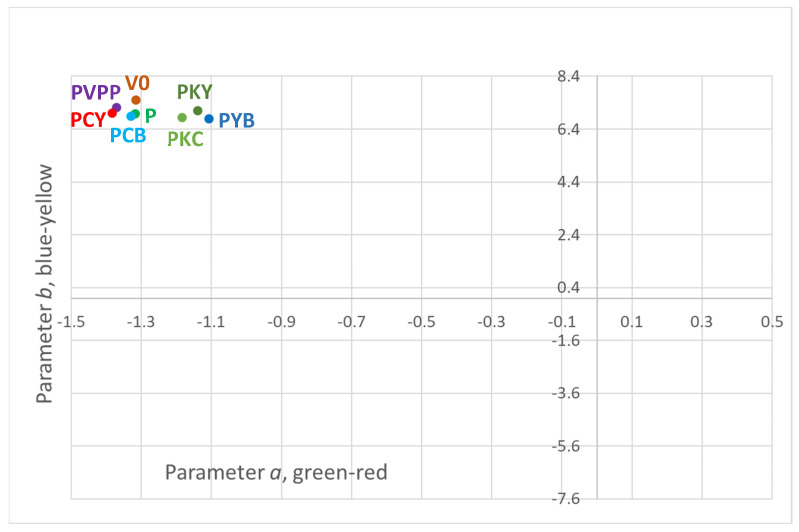
Position of the wine sample colours in the plane formed by the chromaticity coordinates a and b.

These effects on colour parameters are further illustrated in the graphical display of *a* versus *b* values (Figure 1). It can be seen that all are positioned within the yellow–green region of the CIELab chromaticity plane, as defined by the a and b coordinates, with negative *a* values = green and positive *b* = yellow. The samples with the least green tones are PYB, followed by the chitosan-containing samples PKY and PKC, whereas the greenest samples are PVPP and PCY. However, when colour differences (ΔE) were calculated (Table 3), none of the treatments produced a difference considered statistically significant compared to the untreated control (V0), as its colour position falls between those of the treated samples on the colour plane.

The chroma (*c_ab_*, colour saturation) and hue angle (*h_ab_*, hue) reinforce the observations from the *a* and *b* parameters. The highest colour saturation was found in the control sample (*c_ab_* = 7.62), while the hue angles were positioned between 90° (yellow) and 180° (green), closer to yellow, ranging from approximately 99° for PYB and PKY to around 101° for PCB and PCY.

These small differences in colour shades and saturation can be attributed to the removal of certain polyphenols, which in white wines is generally associated with a reduction of red and yellow tones in favour of green. However, in wines treated with ternary combinations containing chitosan and/or yeast hulls (PYB, PKY, and PKC), the shift toward less green tones may also involve additional mechanisms beyond polyphenol removal. The classical PVPP treatment, widely applied in the wine industry to reduce polyphenols in white wines, consistently achieves its intended effect. It produces wines exhibiting some of the greenest colour tones, an effect also observed in PCY, due to the action of activated carbon present in combination. Similar outcomes have been reported for PVPP and active carbon by other authors, who noted comparable reductions in browning in Sherry wines [51].

**Figure 2:**
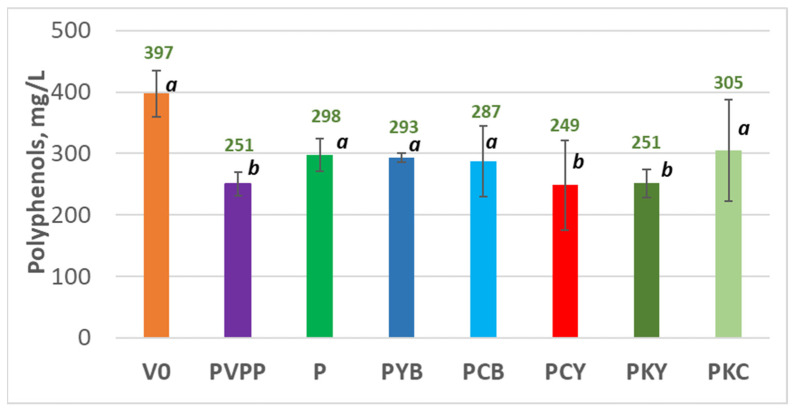
Total polyphenol content of wines not fined (V0) and fined with polyvinylpolypyrrolidone (PV), pea protein (PP), and pea protein combinations with yeast hulls (PKY and PCY). Different letters above the standard deviation bars indicate statistically significant differences among sample means (*p* < 0.05, Tukey’s HSD test), with the letter “a” assigned to the highest mean. Variants sharing at least one letter for a given parameter are not significantly different from each other.

The total polyphenol content (Figure 2) was found to be significantly reduced by the ternary fining combinations containing pea protein and yeast extract (PCY and PKY), as well as by the classical PVPP treatment, with reductions of approximately 37% compared to the control. Other fining agents containing pea protein also decreased the average total polyphenol concentration; however, these reductions (23–28%) were not statistically significant according to the Tukey’s HSD test at *p* < 0.05, likely due to variability among repetitions.

Aside of the total polyphenols, which in white wines include about 80% non-flavonoid compounds, of which 50% hydroxycinnamic acids [52], the content of some flavonoid fractions, such as proanthocyanidins and flavan-3,4-diols, were separately quantified. Proanthocyanidins and flavan-3,4-diols are classes with different structure and degree of polymerization, which can have different gustatory sensory effects. Thus, the concentration of proanthocyanidins influences the sensory balance between bitterness and astringency. A higher degree of polymerization is associated with increased astringency, whereas bitterness tends to peak at a moderate degree of polymerization (approximately DP = 5) [53]. However, our method does not allow for the determination of their degree of polymerization, but provides the concentration of proanthocyanidins, which is not significantly different among the samples including the control.

The highly reactive flavan-3,4-diols, which can polymerize into proanthocyanidins (condensed tannins), exhibit only mild sensory effects as monomers, but contribute a slight astringency and can oxidize to form anthocyanins, imparting red tones even in white wines and thereby reducing the green hue. In our experimental wines, the highest concentration of flavan-3,4-diols was observed in PKC (4.59 mg/L), while other wines treated with pea protein-containing fining agents showed slightly lower, but not significantly different, concentrations (3.76–3.94 mg/L). The lowest flavan-3,4-diol levels were detected in PVPP-treated wines, indicating greater stability to oxidation. Similarly, the sum of proanthocyanidins and flavan-3,4-diols was significantly lower only in the PVPP-treated wines compared to the other samples.

These results suggest that the most effective polyphenol-removing treatments are the ternary combinations containing pea protein and yeast hulls (PCY, PKY), which could serve as viable alternatives to the synthetic fining agent PVPP. To further validate the potential of these treatments as a robust alternative to PVPP, their impact on the aroma quality of the wines was also thoroughly evaluated.

### 4.3. Influence of the Fining Treatments on the Volatile and Aromatic Profile of Wine

The impact of the different fining treatments on the volatile and aromatic composition of the wines was evaluated to determine whether polyphenol removal and other clarifying effects also influenced wine aroma. A comprehensive analysis of key volatile compounds, including alcohols, aldehydes, esters and terpenes, was conducted to assess changes in the aromatic profile and overall sensory quality resulting from the various fining strategies.

The assessment of the volatile profiles of all samples (Table 5), analysed on both chromatographic columns (DB5 and DB1701), revealed that the fining treatments induced notable differences in the concentrations of certain compounds, thereby affecting the final aroma profile of the wines. To interpret these variations, analysis of variance (ANOVA) was performed, and the most significant mean differences are highlighted in bold in Table 5. For discussion purposes, emphasis was placed on the values obtained from the column that exhibited the highest peaks for compounds detected on both columns.

Within the alcohol group, a significant decrease in 2-methylbutan-1-ol of approximately 12.6% was observed in the PKY sample compared to the highest levels recorded in the PVPP-treated wine. Conversely, *cis*-3-hexen-1-ol increased by approximately 37.2%, 21.4%, and 78.0% in PKY, PKC, and PCY, respectively, relative to the control. Additionally, 2-phenylethanol concentrations were higher in the wines treated with yeast hull-containing combinations, showing increases of approximately 53.4% and 131.0% on the DB5 column.

In the aldehyde group, isobutyraldehyde and isovaleraldehyde decreased in both PKY and PCY compared to the control and PVPP-treated wines, by approximately 45.4–45.7% and 6.7–11.4%, respectively, on the DB1701 column. Conversely, 2-phenylacetaldehyde exhibited a marked increase in PKY, PKC, and PCY, with concentrations higher than the control by approximately 336.6%, 125.9%, and 378.8%, respectively, and higher than the PVPP treatment by 226.5%, 68.9%, and 258%, respectively.

In the acetate esters group, butyl acetate decreased while isobutyl acetate increased in the PKY and PCY samples, by approximately 29.3% and 23.7%, and 28.8% and 60%, respectively, compared to the control and PVPP-treated wines. The PKC samples also exhibited a decrease in butyl acetate and a slight increase in isobutyl acetate, although these changes were less pronounced than in PKY and PCY. Isoamyl acetate and hexyl acetate decreased in PCY relative to both the control and PVPP samples, by 7.8% on DB5 and 13.1% on DB1701, respectively. A significant decrease in 2-phenylethyl acetate was observed in wines treated with bentonite-containing combinations, PYB and PCB, with concentrations reduced by 9.8% and 17.9%, respectively, compared to the control.

In the ethyl esters group, the PCY fining combination decreased the concentrations of ethyl formate, ethyl acetate, and ethyl butyrate by approximately 11.2%, 12.1%, and 7.6%, respectively, on the DB5 column. Conversely, ethyl propanoate increased in PKY, PKC, and PCY by approximately 17.3%, 16.9%, and 14.8%, respectively, compared to the control and PVPP-treated samples. Wines treated with ternary combinations containing active carbon (PCB and PCY) exhibited decreases in ethyl octanoate of 12.9% and 10.2% on DB1701, and reductions in ethyl decanoate of approximately 33.0% and 40.5% on DB5.

In the terpenes group, wines treated with yeast hull-containing combinations (PKY and PCY) showed increases in eucalyptol, linalool, and *trans*-linalool oxide of approximately 23.2% and 13.1%, 19.3% and 16.7%, and 341.5% and 428.7%, respectively. In contrast, *β*-myrcene decreased by about 18.9% in PCY relative to the control and by approximately 29.6% compared to PVPP. Ternary fining combinations containing bentonite (PYB and PCB) led to pronounced decreases in linalool and *trans*-linalool oxide, by roughly 17.1% and 18.5%, and 22.2% and 22.7%, respectively. *α*-Ionone increased markedly in the chitosan-containing treatments (PKY and PKC), by 263.0% and 229.5% relative to the control, while all other fined samples also showed significant increases, ranging from 50.7% to 114.0%. These results highlight the important role of sample clarification, regardless of fining agent type, in shaping the final aromatic profile of wines and especially in preserving varietal aroma.

To better interpret these findings and assess whether the increases or decreases in volatile compound concentrations induced by the fining treatments are beneficial, the aroma attributes of the identified compounds were further explained and classified.

Sensory attributes associated with the identified volatile compounds and their anticipated contribution to the aroma profile of the wines are summarized in Table 6. In Table 6, the expected sensory impact of each compound is described, with scores ranging from three minuses (− − −) to one minus (−) for negative aromas, and from one plus (+) to three pluses (+ + +) for positive aromas. Many volatile compounds exhibit a dual sensory role, depending on their concentration: at lower levels they are typically pleasant, while at higher concentrations they may become overwhelming. Exceptions also exist, where even at low concentrations certain compounds are perceived as unpleasant or repulsive; in such cases, they are classified as faults rather than contributors to the aroma profile.

The aliphatic saturated alcohols, such as isobutanol and 2-methylbutan-1-ol, are stable background aromatic compounds that contribute to overall complexity and body [54]. These compounds were only slightly affected by the fining treatments, with a small decrease in 2-methylbutan-1-ol observed for the PKY sample. In contrast, the treatments PKY, PKC, and especially PCY enhanced fresh, herbaceous notes [65], due to marked increases in *cis*-3-hexen-1-ol (up to 78.0% in PCY). Furthermore, the elegant rose and floral character of 2-phenylethanol, a major aroma compound in Muscat wines such as *Tămâ-ioasa românească*, added greater aromatic complexity and enhanced the perception of sweetness in PKY and PCY, with the strongest effect again observed in PCY (131.0%).

Acetaldehyde, typically associated with the negative trait of elevated volatile acidity, was not affected by the treatments. In the low concentrations present, it contributed instead to fresh green apple notes. The Strecker aldehydes isobutyraldehyde [55,56], which are associated with unpleasant oxidative and spoilage aromas, were significantly reduced by PKY and PCY treatments. At the same time, these treatments increased the concentration of 2-phenylacetaldehyde by nearly threefold [57], a pleasant, key floral aroma compound in wines such as *Tămâioasa românească*.

The acetate esters are mostly formed during fermentation, but their concentrations can also be influenced by post-fermentation treatments. Butyl acetate [66], sometimes associated with solvent-like or artificial fruity aromas, decreased in the PKY- and PCY-treated samples. Conversely, isobutyl acetate [67], which shares a similar aromatic profile, increased in the same treatments, contributing to an overall balance in the perceived aroma. Isoamyl acetate, with its well-known banana-like character that can be considered a fault at high levels in aromatic white wines, showed a slight decrease in the PCY samples but was otherwise largely unaffected by fining. Similarly, hexyl acetate [58], which imparts pear-like notes, recorded a small decrease due to PCY treatment. Another compound of high relevance in aromatic wines, 2-phenylethyl acetate [68], is structurally and sensorially related to its alcohol (2-phenylethanol) and aldehyde (2-phenylacetaldehyde) precursors, but contributes a rounder, sweeter, and more honeyed profile, enhancing fruity and floral notes in a smoother, more diffusive way. Unlike its precursors, 2-phenylethyl acetate was negatively affected by treatments containing bentonite, with lower concentrations measured in the PYB and PCB samples.

The ethyl esters, mostly formed during fermentation, are generally responsible for fruity aromas in wines [40,67], with the exception of the smaller-molecule esters, ethyl formate and ethyl acetate, which at higher concentrations are perceived as faults due to their solvent-like character. In the experimental wines, these compounds remained largely unaffected by the fining treatments, except for the PCY combination, which slightly reduced the concentrations of all ethyl esters, including these. PKY and PKC treatments proved beneficial for preserving fruity complexity, particularly pineapple-like notes, by balancing an increase in ethyl propanoate with a slight decrease in ethyl butyrate, while leaving ethyl hexanoate, octanoate, and decanoate unaffected. By contrast, PCY and PCB treatments removed significant amounts of ethyl decanoate and, to a lesser extent, ethyl octanoate, thereby contributing to a reduction in the overall aromatic complexity of the corresponding wines.

Terpenes represent the most characteristic aromatic compounds of Muscat-type wines [27,59], to which *Tămâioasa românească* also belongs. Their contribution is therefore of utmost importance. Linalool [60] plays a key role in enhancing floral and aromatic intensity, often accompanied by its oxidation derivative, *trans*-linalool oxide, which develops during fermentation or aging [61]. Treatments with yeast hull combinations (PKY and PCY) proved particularly beneficial, significantly increasing both compounds and thereby enhancing the lavender and orange blossom notes of linalool, as well as the floral-herbal nuances of *trans*-linalool oxide, the latter showing a threefold increase compared to the control. Fresh, herbal-spicy notes were also reinforced in PKY and PCY through increased eucalyptol [69], also a typical aromatic wine compound [23]. In addition, the subtle violet-like aroma of *α*-ionone, a compound so distinctive that it is sometimes used as a marker of wine authenticity [62], was nearly doubled in chitosan-containing treatments (PKY and PKC). By contrast, bentonite in ternary fining combinations (PYB and PCB) negatively affected the varietal aroma, leading to decreases in both linalool and *trans*-linalool oxide. Finally, PCY reduced the herbal contribution of *β*-myrcene [63], a compound that not only acts as a precursor in terpene biosynthesis, but is also associated with potential health benefits [64].

**Figure 3:**
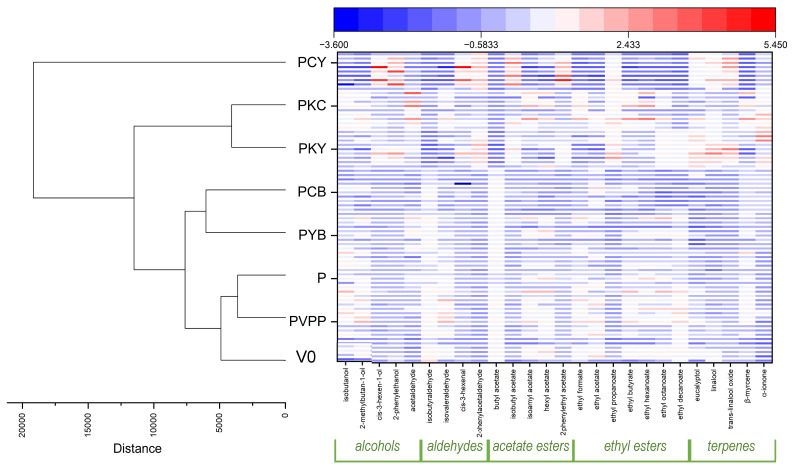
Heatmap (**right**) and cluster analysis (**left**) showing the variations and similarities of volatile compounds in the control and wines treated with PVPP, pea protein and ternary fining agent combinations containing pea protein. Dark blue represents a low relative concentration of the compound in that sample, dark red high relative concentration and intermediate colours gradual increase between low and high values.

To better evaluate the correlation between the applied treatments and the modifications in the volatile profile of the final wine, a heat map was generated and a cluster analysis performed. The heat map (Figure 3, right) clearly shows relatively higher concentrations of several volatile compounds, particularly in the PCY samples, which are also individualized by the cluster analysis (Figure 3, left) as having the most distinct volatile profile compared to the other treatments. Elevated concentrations are likewise evident in samples treated with chitosan-containing fining combinations (PKY and PKC), which cluster closely together, confirming their similar volatile profiles. Among the samples that showed enhanced aromatic profiles, those treated with ternary combinations containing yeast hulls (PCY and PKY) exhibited the highest terpene concentrations.

Samples containing bentonite (PCB and PYB) exhibited many similarities and were grouped together by the cluster analysis, showing profiles characterized by lower concentrations of volatile compounds.

Treatments with pea protein alone and PVPP also led to similar volatile profiles, which were clustered together and found to be the closest to the control samples. This indicates that pea protein, when used as the sole fining agent, represents the most suitable replacement for PVPP in terms of preserving the volatile profile as close as possible to that of untreated wines.

**Figure 4:**
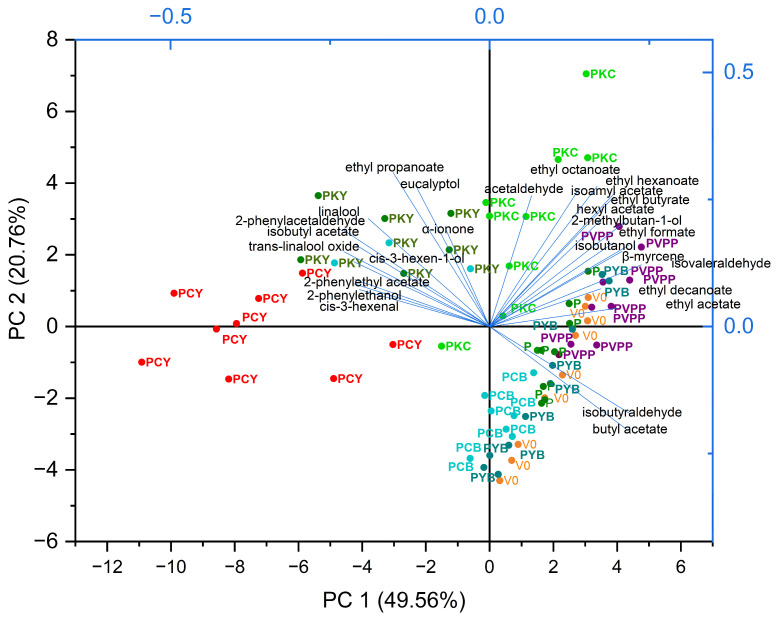
PCA biplot illustrating the differentiation of all wine samples, with nine replicates per sample, based on their volatile profiles.

**Figure 5:**
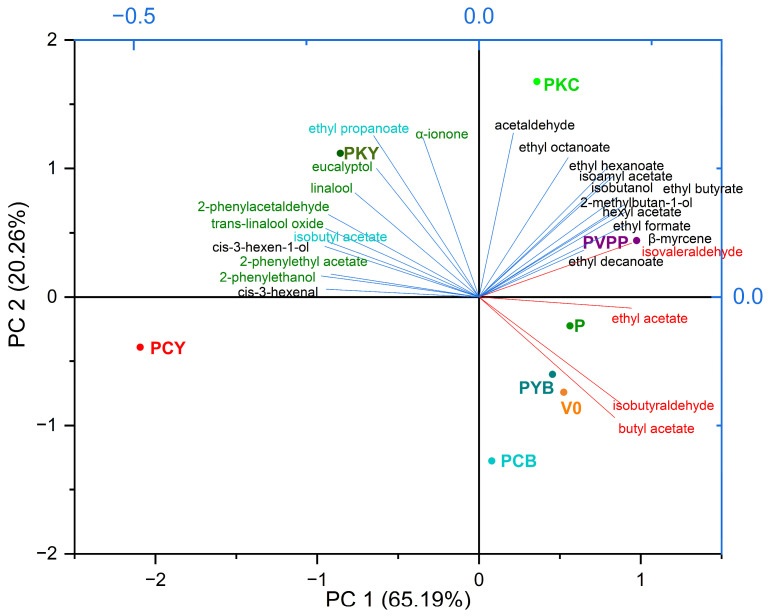
PCA biplot showing the separation of the averaged groups of wine samples according to their volatile profiles. Compounds are color-coded by sensory impact: black (positive aroma), green (positive aroma typical of *Tămâioasa românească*), cyan (dual impact, concentration-dependent), red (negative impact).

Given the multitude of volatile compounds identified and their contribution to the overall aromatic profile, a reduction of variables was carried out using Principal Component Analysis (PCA). In Figure 4, the PCA was applied to all nine replicates for each treatment, while in Figure 5 the mean profile of each treatment group is presented.

In Figure 4, the first two principal components account for 70.32% of the total variance, with PC1 explaining 49.56% and PC2 20.76%. Samples treated with classical PVPP, as well as those treated with pea protein alone (P), clustered closely with the untreated samples (V0), all correlating with fruity ester aromas. In contrast, chitosan-containing treatments were clearly separated in the PCA plot. The PKC treatment tended to preserve an aroma profile dominated by ethyl esters, whereas PKY strongly emphasized varietal characteristics, enhancing compounds associated with Muscat-type aromas. Comparable effects of chitosan fining have also been reported in other studies on white aromatic wines [8]. The PCY treatment was also well separated and correlated with Muscat varietal aromas, though to a lesser extent than PKY. Samples treated with ternary combinations containing bentonite exhibited the simplest aroma profiles, characterized by reduced ester concentrations and a lower abundance of typical Muscat-type compounds. This reduction in volatile aromas has been consistently documented in previous studies, particularly when bentonite is applied during fermentation [70], but also in finished wines, where ester compounds were preferentially removed [71]. These effects are generally attributed to an indirect mechanism, in which bentonite interacts with proteins that in turn bind volatile compounds, thereby reducing their presence in the wine matrix [72].

In Figure 5, the PCA was performed using the average of each wine treatment, allowing a clearer separation of treatments based on the identified volatile compounds. The compounds are color-coded according to their sensory relevance: black for compounds contributing positively to wine aroma; green for compounds strongly associated with the typical aroma of *Tămâioasa românească*; cyan for compounds with dual sensory impact depending on concentration; and red for compounds perceived as having a negative effect on wine aroma. Using averages reduced the variance, and further dimensional reduction via PCA resulted in PC1 covering 65.19% and PC2 20.26% of the total variance, together explaining 85.45%. Based on the calculated coefficients of the extracted eigenvectors (Table 7), it was observed that volatile compounds were distributed across both principal components, with varietal aroma compounds primarily represented on PC1 and fermentation-derived aroma compounds on PC2. This analysis clearly illustrates the negative impact of bentonite and the positive effects of chitosan and yeast hulls on favourable aromas. The PKY combination, containing both chitosan and yeast hulls, produced the most expressive wines, intensifying varietal aromas while preserving fermentation-related aromas.

## 5. Conclusions

The analysis of colour, polyphenols, and volatile composition shows that fining treatments influence both the chemical and sensory profiles of wines. Treatments reducing total polyphenols, such as PVPP and ternary combinations containing pea protein and yeast hulls (PCY, PKY), caused subtle shifts in CIELab parameters, particularly lowering green and yellow hues, and selectively altered flavonoid fractions. PVPP-treated wines had the lowest levels of flavan-3,4-diols and proanthocyanidins, while PCY and PKY achieved comparable polyphenol reductions.

Fining also affected key volatile compounds. PKY and PCY, containing chitosan and yeast hulls, enhanced both varietal and fermentation-derived aromas, increasing terpenes and esters to produce complex, expressive wines. Pea protein alone preserved the aroma profile closest to the untreated control, making it a viable alternative to PVPP. In contrast, bentonite-containing treatments (PCB, PYB) reduced esters and terpenes, simplifying aromas and diminishing varietal character.

A summary of each type of combination effects is the following:

▪ PKY (Pea protein + Chitosan + Yeast hulls): Most expressive wines; high terpenes and esters; floral, fruity, herbal notes.
▪ PKC (Pea protein + Chitosan + Bentonite): Preserves ester-dominated profile; moderate terpene increase; clean and complex wines.
▪ PCY (Pea protein + Chitosan + Yeast hulls): Enhances Muscat-type aromas; slightly reduces some esters; high floral notes.
▪ P (Pea protein alone): Maintains aroma close to untreated; minimal impact on volatiles; PVPP alternative.
▪ PVPP: Effective polyphenol reduction; greener tones; minor aroma changes; less expressive in terpenes.
▪ PCB (Pea protein + Bentonite + Chitosan) & PYB (Pea protein + Yeast hulls + Bentonite): Simplified aromas; reduced esters and terpenes; diminished varietal character.

Overall: Ternary fining combinations with pea protein, particularly those including yeast hulls, effectively manage polyphenols while enhancing aroma complexity, providing a viable alternative to PVPP and optimizing white wine quality.

## 6. Patents: Deposit

A patent application (A/00118/2025) was filed by the University of Agronomic Sciences and Veterinary Medicine Bucharest on March 28, 2025, with OSIM (State Office for Inventions and Trademarks, Romania), entitled “*Tri-component plant-based products based on pea protein for fining white wines*”, authored by Arina Oana Antoce and George Adrian Cojocaru.

## Figures and Tables

**Table 1:** Materials and doses used for fining the experimental wine samples.

Sample Code (Tanks)	PVPP	P/Pea Protein	B/Bentonite	C/Carbon	Y/Yeast Hulls	K/Chitosan
	Dose, g/hL					
V0 (V0_1, V0_2, V0_3)	0	0	0	0	0	0
PVPP (PV_1, PV_2, PV_3)	20	0	0	0	0	0
P (P_1, P_2, P_3)	0	20	0	0	0	0
PYB (PYB_1, PYB_2, PYB_3)	0	10	5	0	5	0
PCB	0	10	5	5	0	0
PCY	0	10	0	5	5	0
PKY	0	10	0	0	5	5
PKC	0	10	0	5	0	5

**Table 2:** Main chemical parameters of the experimental wines one month after the application of the fining treatments.

Sample Code	Free Sugar (Glucose + Fructose), g/L	Ethanol, % *v/v*	Titratable Acidity, g/L	pH	Malic Acid, g/L	Acetic Acid, g/L	Total SO_2_, mg/L
V0	2.22 ± 0.27 ^a^	14.32 ± 0.04 ^a^	4.79 ± 0.06 ^c^	3.6 ± 0.0 ^b^	0.93 ± 0.01 ^a^	0.20 ± 0.07 ^a^	115.67 ± 3.51 ^a^
PVPP	2.03 ± 0.08 ^ab^	14.20 ± 0.11 ^a^	4.84 ± 0.03 ^cb^	3.7 ± 0.1 ^ab^	0.91 ± 0.09 ^a^	0.23 ± 0.01 ^a^	114.67 ± 5.69 ^a^
P	1.76 ± 0.17 ^b^	14.30 ± 0.07 ^a^	4.77 ± 0.02 ^c^	3.7 ± 0.1 ^a^	0.91 ± 0.07 ^a^	0.18 ± 0.08 ^a^	93.00 ± 10.44 ^bc^
PYB	1.75 ± 0.05 ^b^	14.33 ± 0.02 ^a^	4.82 ± 0.01 ^c^	3.7 ± 0.1 ^a^	0.89 ± 0.08 ^a^	0.17 ± 0.08 ^a^	86.00 ± 2.65 ^c^
PCB	1.68 ± 0.09 ^b^	14.22 ± 0.10 ^a^	4.92 ± 0.01 ^ab^	3.7 ± 0.1 ^a^	0.80 ± 0.11 ^a^	0.19 ± 0.10 ^a^	101.00 ± 2.59 ^abc^
PCY	1.77 ± 0.09 ^b^	14.24 ± 0.08 ^a^	4.92 ± 0.02 ^ab^	3.7 ± 0.1 ^ab^	0.73 ± 0.01 ^a^	0.22 ± 0.01 ^a^	99.67 ± 6.03 ^abc^
PKY	1.96 ± 0.08 ^ab^	14.24 ± 0.08 ^a^	4.98 ± 0.02 ^a^	3.6 ± 0.1 ^b^	0.81 ± 0.09 ^a^	0.22 ± 0.01 ^a^	101.67 ± 3.21 ^abc^
PKC	1.89 ± 0.09 ^ab^	14.31 ± 0.01 ^a^	4.97 ± 0.04 ^a^	3.6 ± 0.1 ^b^	0.73 ± 0.06 ^a^	0.29 ± 0.03 ^a^	109.33 ± 10.97 ^ab^

Average values ± standard deviations (*n* = 3). Different letters within the same row indicate statistically significant differences among sample means (*p* < 0.05, Tukey’s HSD test), with the letter “a” always assigned to the highest mean. Variants sharing at least one letter for a given parameter are not significantly different from each other.

**Table 3:** CIELab colour parameters of the experimental wines one month after the application of the fining treatments.

Sample Code	*L*	*a*	*b*	*c_ab_*	*h_ab_*	Δ*E*
V0	98.18 ± 0.22 ^ab^	−1.32 ± 0.08 ^abc^	7.50 ± 0.04 ^a^	7.62 ± 0.04 ^a^	100.04 ± 0.01 ^abc^	
PVPP	97.80 ± 0.15 ^b^	−1.37 ± 0.03 ^a^	7.22 ± 0.03 ^ab^	7.35 ± 0.03 ^ab^	100.86 ± 0.00 ^abc^	0.52 ± 0.20 ^a^
P	98.41 ± 0.08 ^a^	−1.32 ± 0.07 ^ab^	6.98 ± 0.24 ^bcd^	7.11 ± 0.25 ^bcd^	100.82 ± 0.00 ^abc^	0.62 ± 0.15 ^a^
PYB	98.25 ± 0.18 ^ab^	−1.11 ± 0.15 ^c^	6.78 ± 0.10 ^d^	6.87 ± 0.07 ^d^	99.22 ± 0.02 ^bc^	0.82 ± 0.03 ^a^
PCB	98.51 ± 0.17 ^a^	−1.33 ± 0.04 ^ab^	6.88 ± 0.09 ^cd^	7.01 ± 0.09 ^cd^	101.09 ± 0.00 ^ab^	0.76 ± 0.08 ^a^
PCY	98.40 ± 0.18 ^a^	−1.38 ± 0.02 ^a^	7.00 ± 0.03 ^bcd^	7.13 ± 0.03 ^abc^	101.24 ± 0.00 ^a^	0.59 ± 0.05 ^a^
PKY	98.44 ± 0.09 ^a^	−1.14 ± 0.02 ^bc^	7.11 ± 0.06 ^bc^	7.20 ± 0.06 ^bc^	99.09 ± 0.00 ^c^	0.52 ± 0.05 ^a^
PKC	98.60 ± 0.14 ^a^	−1.18 ± 0.07 ^abc^	6.83 ± 0.01 ^cd^	6.93 ± 0.02 ^cd^	99.87 ± 0.01 ^abc^	0.83 ± 0.06 ^a^

Average values ± standard deviations (*n* = 3). Different letters within the same row indicate statistically significant differences among sample means (*p* < 0.05, Tukey’s HSD test), with the letter “a” always assigned to the highest mean. Variants sharing at least one letter for a given parameter are not significantly different from each other.

**Table 4:** Estimation of some polyphenol classes present in the experimental wines one month after the application of the fining treatments.

Sample Code	Proanthocyanidins	Flavan-3,4-diols	Sum Proanthocyanidins+Flavan-3,4-diols	Total Polyphenols, mg/L	Total Polyphenols, %
	mg/L Cyanidin Equivalents				
V0	15.12 ± 0.57 ^a^	3.70 ± 0.51 ^c^	18.82 ± 0.08 ^ab^	397.33 ± 37.50 ^a^	100.00
PVPP	12.27 ± 0.16 ^a^	2.59 ± 0.33 ^b^	14.87 ± 0.03 ^b^	251.00 ± 19.16 ^b^	63.17
P	15.30 ± 1.49 ^a^	3.94 ± 0.12 ^ac^	19.24 ± 1.48 ^ab^	297.67 ± 26.10 ^ab^	74.92
PYB	15.63 ± 0.29 ^a^	3.87 ± 0.53 ^ac^	19.50 ± 0.39 ^ab^	293.00 ±7.21 ^ab^	73.74
PCB	16.65 ± 1.15 ^a^	3.76 ± 0.19 ^ac^	20.41 ± 1.17 ^a^	286.67 ± 57.55 ^ab^	72.15
PCY	15.54 ± 0.24 ^a^	3.84 ± 0.33 ^ac^	19.38 ± 0.51 ^ab^	248.67 ± 73.22 ^b^	62.59
PKY	15.99 ± 4.61 ^a^	3.94 ± 0.18 ^ac^	19.92 ± 4.47 ^a^	251.33 ± 22.37 ^b^	63.26
PKC	14.86 ± 1.21 ^a^	4.59 ± 0.05 ^a^	19.45 ± 0.1.23 ^ab^	305.33 ± 82.25 ^ab^	76.85

Average values ± standard deviations (*n* = 3). Different letters within the same row indicate statistically significant differences among sample means (*p* < 0.05, Tukey’s HSD test), with the letter “a” always assigned to the highest mean. Variants sharing at least one letter for a given parameter are not significantly different from each other.

**Table 5:** Volatile compounds identified in the experimental wines one month after fining treatments, expressed as peak areas from chromatograms obtained on two columns of different polarity (DB5 and DB1701).

Adj. R^2^	Column	Identified Compounds	Weighted Kovats Index	Control	PVPP	P	PYB	PCB	PKY	PKC	PCY
***Alcohols***											
0.573	DB5	**isobutanol**	624.8	7378.55 ± 448.64 ^bcd^	8135.19 ± 275.00 ^a^	7818.19 ± 320.37 ^abc^	7574.70 ± 103.32 ^bc^	7324.14 ± 325.61 ^cd^	7364.14 ± 138.10 ^d^	7932.53 ± 273.83 ^ab^	6831.46 ± 500.72 ^d^
0.473	DB5	**2-methylbutan-1-ol**	739.8	166,103.08 ± 7539.16 ^abc^	176,903.50 ± 6346.12 ^a^	170,950.69 ± 4625.53 ^abc^	168,064.87 ± 8315.51 ^abc^	163,699.88 ± 7197.66 ^abc^	163,016.53 ± 6167.99 ^bcd^	174,563.15 ± 6840.33 ^cd^	**154,644.89 ± 5796.10 ^d^**
0.505	DB1701		849.9	132,524.24 ± 1991.44 ^bcd^	138,097.00 ± 2234.83 ^a^	135,731.87 ± 4073.72 ^abc^	131,498.57 ± 5558.89 ^abcde^	129,153.50 ± 4543.37 ^cde^	128,651.14 ± 4499.21 ^de^	136,622.50 ± 4080.49 ^ab^	**123,949.93 ± 5349.94 ^e^**
0.475	DB1701	***cis*** **-3-hexen-1-ol**	969.2	2821.27 ± 188.96 ^c^	3065.27 ± 204.18 ^abc^	2952.47 ± 160.75 ^abc^	2952.90 ± 140.50 ^abc^	2912.74 ± 219.22 ^bc^	**3870.18 ± 740.59 ^ab^**	3425.84 ± 356.78 ^a^	**5020.75 ± 1766.82 ^abc^**
0.518	DB5	**2-phenylethanol**	1107.2	779.39 ± 85.31 ^abc^	707.29 ± 28.53 ^bc^	698.57 ± 50.90 ^bc^	691.49 ± 50.06 ^c^	665.01 ± 91.54 ^bc^	**1195.85 ± 495.17 ^abc^**	788.23 ± 65.89 ^ab^	**1800.71 ± 824.65 ^a^**
0.741	DB1701		1276.9	682.14 ± 28.65 ^cde^	714.95 ± 28.74 ^bcd^	717.33 ± 66.10 ^bcde^	655.70 ± 34.12 ^e^	658.19 ± 77.50 ^de^	**1685.11 ± 766.25 ^abc^**	809.01 ± 74.45 ^b^	**1879.85 ± 37.11 ^a^**
***Aldehydes***											
0.550	DB1701	**acetaldehyde**	547.1	4673.70 ± 145.63 ^d^	5107.07 ± 275.02 ^bc^	5252.23 ± 361.86 ^abc^	5519.60 ± 490.73 ^abc^	4859.69 ± 227.12 ^cd^	5305.19 ± 148.01 ^ab^	6258.78 ± 849.25 ^a^	4886.67 ± 254.63 ^cd^
0.905	DB1701	**isobutyraldehyde**	819.8	779.33 ± 71.96 ^ab^	799.49 ± 33.53 ^a^	707.73 ± 40.38 ^b^	770.81 ± 52.92 ^ab^	739.47 ± 61.98 ^ab^	**425.40 ± 42.97 ^d^**	563.13 ± 36.36 ^c^	**418.40 ± 28.66 ^d^**
0.694	DB5	**isovaleraldehyde** (3-methylbutanal)	653.9	641.18 ± 40.79 ^a^	637.63 ± 69.62 ^abc^	603.98 ± 10.38 ^ab^	578.78 ± 20.30 ^bc^	550.23 ± 33.34 ^cd^	**472.15 ± 68.43 ^de^**	576.99 ± 17.55 ^c^	**422.94 ± 69.28 ^e^**
0.646	DB1701		731.1	11,267.74 ± 504.46 ^abc^	11,966.26 ± 470.64 ^a^	11,468.16 ± 260.32 ^ab^	11,090.87 ± 322.54 ^bc^	10,748.97 ± 209.38 ^c^	**10,516.14 ± 540.93 ^cd^**	11,500.81 ± 444.31 ^ab^	**9979.41 ± 538.42 ^d^**
0.419	DB5	***cis*** **-3-hexenal**	786	276.83 ± 59.87 ^ab^	337.12 ± 67.01 ^ab^	295.34 ± 18.87 ^b^	329.43 ± 21.10 ^a^	261.76 ± 141.59 ^ab^	451.86 ± 197.96 ^ab^	328.37 ± 112.86 ^ab^	804.59 ± 441.34 ^ab^
0.914	DB5	**2-phenylacetaldehyde**	1042.4	56.92 ± 2.19 ^d^	76.12 ± 8.58 ^c^	81.27 ± 13.01 ^c^	70.04 ± 17.90 ^cd^	72.36 ± 11.33 ^c^	**248.53 ± 50.88 ^a^**	**128.59 ± 28.26 ^b^**	**272.52 ± 28.67 ^a^**
Acetate Esters *											
0.954	DB5	**butyl acetate**	817.6	644.55 ± 25.26 ^a^	656.06 ± 59.97 ^a^	587.40 ± 75.67 ^a^	643.49 ± 13.78 ^a^	617.83 ± 36.75 ^a^	**188.82 ± 53.73 ^c^**	**297.86 ± 44.82 ^b^**	**152.83 ± 10.49 ^c^**
0.622	DB5	**isobutyl acetate** (isobutyl ethanoate)	771.0	1518.54 ± 112.94 ^b^	1681.06 ± 104.44 ^b^	1579.96 ± 64.75 ^b^	1618.38 ± 103.56 ^b^	1613.96 ± 116.34 ^b^	**1956.59 ± 173.35 ^a^**	1833.73 ± 280.31 ^ab^	**2440.52 ± 460.95 ^a^**
0.461	DB5	**isoamyl acetate** (3-methylbutyl ethanoate)	870.5	25,778.37 ± 1262.69 ^abc^	27,037.34 ± 1042.26 ^a^	25,934.23 ± 780.43 ^abc^	25,572.72 ± 1546.96 ^abcd^	24,959.43 ± 374.18 ^c^	25,239.64 ± 665.52 ^bc^	27,204.52 ± 1483.81 ^ab^	**23,777.46 ± 806.27 ^d^**
0.422	DB1701		938.5	17,870.12 ± 845.94 ^ab^	18,508.53 ± 329.44 ^a^	17,974.11 ± 439.96 ^ab^	17,650.99 ± 1085.55 ^abc^	17,509.82 ± 460.71 ^b^	17,552.60 ± 393.41 ^b^	18,697.83 ± 942.16 ^ab^	**16,569.84 ± 494.68 ^c^**
0.270	DB5	**hexyl acetate**	1003.7	473.02 ± 42.44 ^a^	477.93 ± 40.07 ^a^	455.35 ± 35.82 ^a^	449.43 ± 57.84 ^ab^	401.10 ± 17.73 ^b^	433.48 ± 51.74 ^ab^	465.70 ± 66.39 ^ab^	**384.87 ± 44.50 ^b^**
0.347	DB1701		1075.5	675.59 ± 35.74 ^ab^	716.75 ± 24.42 ^a^	675.36 ± 40.44 ^ab^	672.13 ± 67.95 ^abc^	645.55 ± 12.23 ^b^	653.91 ± 33.96 ^b^	691.22 ± 79.29 ^abc^	**587.44 ± 25.17 ^c^**
0.599	DB1701	**2-phenylethyl acetate**	1338.4	132.24 ± 10.86 ^cd^	140.49 ± 3.78 ^bc^	147.87 ± 19.92 ^bc^	**119.28 ± 12.30 ^de^**	**108.54 ± 6.53 ^e^**	186.93 ± 20.31 ^a^	133.96 ± 21.76 ^cde^	286.70 ± 114.11 ^ab^
***Ethyl esters***											
0.677	DB5	**ethyl formate**	541.5	76,684.69 ± 2167.09 ^a^	79,766.50 ± 2428.92 ^a^	76,971.01 ± 2406.17 ^a^	75,683.30 ± 4391.43 ^ab^	72,918.82 ± 989.62 ^b^	72,132.80 ± 3047.97 ^bc^	80,351.78 ± 2682.08 ^a^	**68,099.56 ± 1157.63 ^c^**
0.631	DB1701		582.5	58,655.02 ± 3193.07 ^abc^	60,749.90 ± 1630.88 ^a^	58,176.74 ± 1584.48 ^ac^	57,264.03 ± 3041.25 ^abc^	55,389.34 ± 1079.34 ^b^	54,742.72 ± 2623.20 ^bcd^	60,528.22 ± 1318.58 ^a^	**52,082.11 ± 720.40 ^d^**
0.423	DB5	**ethyl acetate**	614.5	79,097.53 ± 3293.11 ^a^	79,651.64 ± 3565.31 ^a^	77,027.24 ± 3212.75 ^a^	75,415.68 ± 3322.98 ^a^	78,270.62 ± 3842.38 ^a^	73,778.59 ± 4243.77 ^ab^	77,349.69 ± 3676.11 ^a^	**69,544.97 ± 1779.45 ^b^**
0.411	DB1701		677	58,400.27 ± 2495.85 ^a^	59,381.77 ± 2666.82 ^a^	57,276.20 ± 2006.80 ^a^	56,178.44 ± 2147.81 ^a^	57,882.72 ± 2899.98 ^a^	54,326.62 ± 3466.29 ^ab^	56,922.87 ± 2582.70 ^a^	**51,635.60 ± 2427.58 ^b^**
0.794	DB1701	**ethyl propanoate**	769.5	2049.62 ± 86.81 ^bc^	2086.04 ± 63.24 ^b^	2076.47 ± 93.43 ^bc^	1975.80 ± 46.58 ^c^	2049.46 ± 88.74 ^bc^	**2404.57 ± 139.56 ^a^**	**2396.40 ± 52.80 ^a^**	**2352.71 ± 66.34 ^a^**
0.510	DB5	**ethyl butyrate**	796.4	5587.00 ± 267.24 ^ab^	5867.52 ± 204.89 ^a^	5662.63 ± 133.37 ^ab^	5508.65 ± 237.58 ^ab^	5520.06 ± 188.29 ^b^	5545.20 ± 53.43 ^b^	5919.55 ± 299.50 ^ab^	**5160.94 ± 171.00 ^c^**
0.308	DB5	**ethyl hexanoate**	991.3	15,758.77 ± 870.79 ^abc^	16,456.57 ± 531.86 ^a^	16,059.06 ± 716.57 ^ab^	15,814.54 ± 1255.01 ^abcd^	15,001.87 ± 224.11 ^cd^	15,585.15 ± 304.65 ^b^	16,668.46 ± 1810.23 ^abcd^	**14,469.61 ± 545.79 ^d^**
0.345	DB1701		1056.9	11,805.61 ± 623.82 ^abc^	12,394.14 ± 452.99 ^a^	12,102.01 ± 526.59 ^ab^	11,809.79 ± 842.76 ^abcd^	11,271.09 ± 140.59 ^cd^	11,767.17 ± 117.67 ^b^	12,672.19 ± 1374.69 ^abcd^	10,908.74 ± 412.80 ^d^
0.620	DB5	**ethyl octanoate**	1189.9	15,540.08 ± 912.89 ^ab^	16,305.46 ± 1133.47 ^ab^	15,331.32 ± 726.07 ^b^	15,492.52 ± 1453.57 ^ab^	**13,403.70 ± 520.27 ^c^**	16,477.86 ± 313.67 ^a^	15,774.70 ± 976.63 ^ab^	**13,157.18 ± 496.45 ^c^**
0.453	DB1701		1257.6	20,435.58 ± 1186.61 ^a^	21,190.73 ± 951.65 ^a^	20,243.18 ± 982.92 ^ab^	20,510.42 ± 1978.66 ^ab^	**17,791.17 ± 528.09 ^c^**	21,437.83 ± 603.11 ^a^	20,334.01 ± 1803.20 ^ab^	**18,354.10 ± 1300.93 ^bc^**
0.953	DB5	**ethyl decanoate**	1385	5277.23 ± 130.54 ^b^	5728.74 ± 183.03 ^a^	4740.93 ± 28.39 ^c^	4927.96 ± 370.01 ^bc^	**3535.42 ± 132.35 ^e^**	5114.56 ± 107.39 ^b^	4045.32 ± 179.04 ^d^	**3140.32 ± 182.13 ^f^**
0.933	DB1701		1453.6	3736.40 ± 191.63 ^ab^	3906.41 ± 133.60 ^a^	3114.83 ± 111.58 ^c^	3312.50 ± 325.79 ^bc^	**2222.63 ± 124.49 ^e^**	3642.46 ± 100.24 ^b^	2665.27 ± 125.57 ^d^	**2120.16 ± 156.77 ^e^**
***Terpenes and derivates***											
0.615	DB5	**eucalyptol** (1,8-cineole)	1026.5	310.71 ± 32.62 ^bcde^	325.28 ± 18.70 ^bc^	296.45 ± 15.63 ^de^	269.23 ± 27.41 ^e^	292.73 ± 34.44 ^cde^	**382.85 ± 9.17 ^a^**	325.12 ± 31.25 ^bcd^	**351.40 ± 26.15 ^ab^**
0.805	DB1701	**linalool**	1191.7	309.29 ± 19.41 ^b^	282.48 ± 9.95 ^cd^	266.77 ± 15.08 ^de^	**256.28 ±** **8.73 ^e^**	**252.20 ±** **4.05 ^e^**	**368.90 ± 37.28 ^a^**	309.55 ± 22.30 ^bc^	**361.04 ± 26.98 ^a^**
0.633	DB5	***trans*** **-linalool oxide**	1091.3	196.46 ± 20.73 ^c^	190.06 ± 37.54 ^cd^	217.28 ± 56.55 ^cd^	**152.92 ± 10.44 ^d^**	**151.82 ± 5.58 ^d^**	**867.30 ± 475.25 ^ab^**	359.18 ± 38.19 ^b^	**1038.75 ± 500.89 ^a^**
0.590	DB1701	***β-*** **myrcene**	1015.1	198.73 ± 13.08 ^b^	228.77 ± 8.62 ^a^	216.83 ± 17.27 ^ab^	206.03 ± 15.88 ^b^	210.73 ± 2.44 ^b^	192.04 ± 19.83 ^b^	231.39 ± 34.00 ^ab^	**161.02 ± 1.01 ^c^**
0.668	DB5	***α*** **-ionone**	1426.3	362.51 ± 137.05 ^e^	578.37 ± 173.33 ^cde^	546.38 ± 66.89 ^de^	698.28 ± 97.53 ^c^	775.69 ± 77.08 ^bc^	**1316.03 ± 451.36 ^ab^**	**1194.48 ± 234.66 ^a^**	721.93 ± 165.33 ^bcd^

Chromatographic data are expressed as mean peak areas (*n* = 9) ± standard deviation. Data were analysed using Welch’s ANOVA followed by the Games–Howell post hoc test. Different letters within the same row indicate significant differences between sample means, with “a” always assigned to the highest mean. Values shown in bold font highlight particularly important differences, which are discussed in detail in Section 4. * Ethyl acetate was introduced in the class of ethyl esters.

**Table 6:** Aroma descriptors of identified volatile compounds and their expected sensory impact in wine.

Identified Volatile Compounds	Aroma Descriptors *	Impact on Wine Aroma	
		*Lower Concentrations*	*Higher Concentrations*
*Alcohols*			
**isobutanol**	solvent, bitter, glue, alcohol, leek, licorice	+ enhances the overall complexity	− − − chemical off-odors, adding a solvent-like or alcoholic note
**2-methylbutan-1-ol**	malty, cooked, roasted aroma with fruity or alcoholic undernotes, buttery	+ enhances complexity or body	− − − solvent-like, nail polish remover notes, considered a fault
***cis*** **-3-hexen-1-ol**	fresh, green, grassy, leafy	+ + fresh, herbaceous complexity	− − overly grassy, green, overpowering more delicate aromas
**2-phenylethanol**	floral, rose-like aroma, llilac, herbal spicy, honey-like, sweet, yeast	+ + + elegant rose and floral notes adds complexity, enhances the perceived sweetness	
*Aldehydes*			
**acetaldehyde**	green apple, cut grass, nutty, sherry-like, bruised apple flavour, paint	+ green apple notes, nutty-oxidative aroma	− − − paint, pungent, oxidized, considered a fault
**isobutyraldehyde**	green, pungent, burnt, malty, toasted, fruity	+/− contributes to perceived graininess or maltiness, may even be perceived as a positive character	− − − undesirable aromas, associated with oxidation or spoilage. In young wines can reduce fruitiness.
**Isovaleraldehyde** (3-methylbutanal)	fruity, almond, toasted, malty, green, herbal	+ pleasant, complex aromas, contributes to wine’s bouquet	− − unpleasant aromas described as “cardboard”, “rancid”, “ cheesy” or “sweaty”
***cis*** **-3-hexenal**	freshly cut grass and leaves	+ + pleasant freshly cut green apple	− − raw, vegetal, or unripe
**2-phenylacetaldehyde**	floral, rose, lilac, hyacinth, sweet, honey-like and slightly fruity	+ + + pleasant, key aroma compound in many floral wines	− heavy, perfumy character
Acetate Esters *			
**butyl acetate**	fruity, banana, pear, pineapple, bitter, green, sweaty, strong	+ reminiscent of red delicious apples	− − solvent-like, artificial, or nail-polish remover-like, fault
**isobutyl acetate** (isobutyl ethanoate)	fruity, pear, banana	+ fruity pear-banana scent reminiscent of raspberry	− − solvent-like, artificial, glue-like, fault
**isoamyl acetate** (3-methylbutyl ethanoate)	banana-like or fruity aroma, fresh, sweet, fruity, pear	+ + pleasant banana, pear and bubblegum aroma	− − synthetic or chemical odor
**hexyl acetate**	fruity, green, grassy aroma, spicy, herbal, rubbery, tobacco, citrus	+ + described as pear-like with floral notes	− overly green or herbaceous
**2-phenylethyl acetate**	rose, floral, fruity, sweet, sometimes rasppery/peach aroma	+ + + desirable pleasant floral and fruity aroma	
*Ethyl esters*			
**ethyl formate**	rum-like and sometimes fruity aroma, particularly resembling raspberries, sweet	+ + ethereal-fruity	− solvent-like, artificial, or nail-polish remover-like, fault
**ethyl acetate**	caramel, sweet, solvent, fruity, acid, buttery, pungent	+ adds fruity, fresh notes	− − overpowering, solvent-like, a sign of acetic spoilage, fault
**ethyl propanoate**	fruity, apple, pear, pineapples, sweet, solvent, acetone	+ + sweet aroma reminiscent of pineapples and pears	− rarely overly sweet or artificial
**ethyl butyrate**	tropical fruit, overripe bananas, pineapple, caramel	+ + sweet fruity notes	− rarely overly sweet or artificial
**ethyl hexanoate**	apples, pineapple, fruity, strawberry, anise, sweet, green apple, slightly floral	+ + fresh, fruity notes, enhances overall aroma complexit	− rarely overly sweet, heavy, or slightly solvent-like
**ethyl octanoate**	fruity, pear, apricot, banana, pineapple, fresh, floral, fatty, green, leafy, anise, baked fruit, sweet, soapy	+ + fruity, pleasant, enhances fruity complexity	− overly sweet, heavy, or slightly fatty
**ethyl decanoate**	sweet, apple, pear, and brandy-like aroma, grape, fruity	+ + contributes to roundness, complexity, and subtle fruity notes	− fatty or soapy
*Terpenes and derivates*			
**eucalyptol** (1,8-cineole)	herbal, camphor-like, eucalyptus spicy, mint, cooling	+ + adds complexity, freshness, and herbal/spicy nuance	− medicinal, camphor-like
**linalool**	floral and slightly citrus aroma, flowery, lavender, orange blossom	+ + + enhances floral and aromatic intensity	− overly perfumed or artificial
***trans*** **-linalool oxide**	pleasant floral and herbal scent, sweet, floral, creamy, leafy, earthy, green	+ + + adds complexity and soft floral nuances	rarely a problem
***β-*** **myrcene**	herbal, resinous, anise, grape, fruity, herbaceous, vanilla, wine-like, vegetable, woody, green, metallic, musty, geranium, sweet, ethereal, soapy, lemon, spicy	+ + subtle herbal, resinous, citrusy aroma, enhancing complexity	− too green, resinous or medicinal
***α*** **-ionone**	violet, floral, and sometimes woody aroma	+ + + adds subtle floral and violet notes, enhancing complexity	

* Aroma descriptors for the identified volatile compounds were sourced from well-established databases [42,43,44], supplemented by findings from our previous research [10,40,41,45], peer-reviewed scientific literature [54,55,56,57,58,59,60,61,62,63,64], and online resources focusing on the sensory impact of volatile compounds [65,66,67,68,69].

**Table 7:** Loadings of volatile compounds on principal components obtained from PCA of averaged wine sample groups.

Volatile Compounds	Loading on PC1 65.19%	Loadings on PC2 20.26%
Positive aroma varietal compounds		
*cis*-3-hexen-1-ol	0.24351	0.1515
2-phenylethanol	0.24576	−0.01736
2-phenylacetaldehyde	0.28406	0.07289
2-phenylethyl acetate	0.22382	0.04395
eucalyptol	0.19756	0.15424
linalool	0.25785	0.0217
*trans*-linalool oxide	0.27522	0.01049
β-myrcene	−0.12992	0.27925
α-ionone	0.18613	0.11434
Positive aroma fermentation compounds		
isobutanol	−0.11392	0.24584
2-methylbutan-1-ol	−0.1528	0.28321
acetaldehyde	0.06346	0.25489
*cis*-3-hexenal	0.19369	0.10026
isoamyl acetate	−0.11493	0.32901
hexyl acetate	−0.12212	0.23973
ethyl formate	−0.18579	0.21936
ethyl butyrate	−0.08848	0.33594
ethyl hexanoate	−0.06248	0.31162
ethyl octanoate	0.06949	0.233
ethyl decanoate	−0.07229	−0.07601
Negative or dual aroma compounds		
isobutyl acetate	0.21601	0.18456
ethyl propanoate	0.22727	0.18809
ethyl acetate	−0.18322	0.0738
isobutyraldehyde	−0.26687	−0.03865
isovaleraldehyde	−0.22244	0.20702
butyl acetate	−0.26549	−0.07573

## Data Availability

The original contributions presented in this study are included in the article. The raw data supporting the conclusions of this article will be made available by the authors on request.
